# A Self-Learning Hyper-Heuristic Algorithm Based on a Genetic Algorithm: A Case Study on Prefabricated Modular Cabin Unit Logistics Scheduling in a Cruise Ship Manufacturer

**DOI:** 10.3390/biomimetics9090516

**Published:** 2024-08-27

**Authors:** Jinghua Li, Ruipu Dong, Xiaoyuan Wu, Wenhao Huang, Pengfei Lin

**Affiliations:** 1 College of Mechanical and Electrical Engineering, Harbin Engineering University, Harbin 150001, China; 2 Sanya Nanhai Innovation and Development Base of Harbin Engineering University, Harbin Engineering University, Sanya 572024, China; 3 College of Shipbuilding Engineering, Harbin Engineering University, Harbin 150001, China; 4 Shanghai Waigaoqiao Shipbuilding Co., Ltd., Shanghai 200137, China

**Keywords:** self-learning hyper-heuristic algorithm based on genetic algorithm, fuzzy logistics scheduling, cruise ship, PMCU

## Abstract

Hyper-heuristic algorithms are known for their flexibility and efficiency, making them suitable for solving engineering optimization problems with complex constraints. This paper introduces a self-learning hyper-heuristic algorithm based on a genetic algorithm (GA-SLHH) designed to tackle the logistics scheduling problem of prefabricated modular cabin units (PMCUs) in cruise ships. This problem can be regarded as a multi-objective fuzzy logistics collaborative scheduling problem. Hyper-heuristic algorithms effectively avoid the extensive evaluation and repair of infeasible solutions during the iterative process, which is a common issue in meta-heuristic algorithms. The GA-SLHH employs a genetic algorithm combined with a self-learning strategy as its high-level strategy (HLS), optimizing low-level heuristics (LLHs) while uncovering potential relationships between adjacent decision-making stages. LLHs utilize classic scheduling rules as solution support. Multiple sets of numerical experiments demonstrate that the GA-SLHH exhibits a stronger comprehensive optimization ability and stability when solving this problem. Finally, the validity of the GA-SLHH in addressing real-world decision-making issues in cruise ship manufacturing companies is validated through practical enterprise cases. The results of a practical enterprise case show that the scheme solved using the proposed GA-SLHH can reduce the transportation time by up to 37%.

## 1. Introduction

Meta-heuristic algorithms are a class of algorithms used to solve complex optimization problems through searching and iteration to find the optimal or sub-optimal solution to the problem. This kind of algorithm has been widely used in various optimization problems (such as shop scheduling, path planning, service scheduling, etc.) because of its practicability and generality. Currently, meta-heuristic algorithms can be divided into evolutionary-based algorithms, bio-based algorithms, human-based algorithms, physics-based algorithms, math-based algorithms, and other algorithms [1,2]. However, when the meta-heuristic algorithm deals with optimization problems with some complex constraints, there are some shortcomings in the flexibility of operation in an individual iteration. Hyper-heuristic algorithms offer significant advantages in solving complex optimization problems [3,4]. Their primary strength lies in their ability to dynamically select or generate heuristics during the problem-solving process, leading to more efficient and effective solutions [5]. This flexibility allows hyper-heuristic algorithms to adapt to a wide range of problem types and constraints, making them highly applicable in various fields, including decision making, manufacturing, and logistics [6,7]. This feature enables the efficient scheduling of the prefabricated modular cabin unit (PMCU) logistics process with complex processes and task interactions. This occurs very frequently in cruise ship manufacturers but lacks an effective scheduling scheme to guide the process.

**Figure 1:**
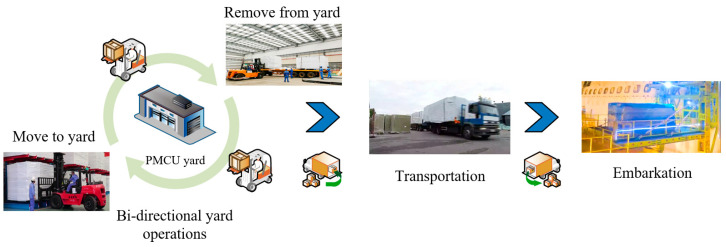
Three stages of PMCU logistics.

The prefabricated modular cabin unit (PMCU) is currently the most popular technology used by cruise ship manufacturers for the construction of large numbers of cabins [8]. Assembly in the workshop and transportation on board are the logistical processes that manufacturers have to go through in order to adopt this technology [9,10]. A diagram of PMCU logistics within the shipyard is shown in Figure 1. Once the PMCUs have been assembled, they will be moved from the assembly line into a temporary yard, transported by delivery trucks to the shipyard as required, and then transported on board the ship via a specialized cabin lift. The cabin lift also undertakes the transportation process for outfitting supply embarkation. With multiple types of embarkation tasks running in parallel, the impact of multi-task occupancy also needs to be taken into account when establishing the PMCU embarkation plan. It can be seen that the yard operations and transportation of PMCUs have well-connected logistics. PMCU logistics comprises a series of mutual influences and mutual constraints of the operation process, requiring various types of equipment and resources. As can be seen, PMCU logistics needs a rational and effective production scheme to guide its activities. Unfortunately, domestic manufacturers are not able to effectively schedule this logistical process, leading to actual transportation inefficiency and a lack of ability to meet the planned expectations of the cruise project. Therefore, the aim of this study is to develop and introduce a novel and effective scheduling method based on a hyper-heuristic framework for optimizing cruise ships.

Unlike the highly automated logistics industry, the shipbuilding industry belongs to the large-scale structure and equipment manufacturing industry in which the transportation of intermediate products is less automated and standardized [11]. For the logistics of the PMCUs of cruise ships, yard operations and transportation collaboration processes have the following characteristics: (1) adopting Just-In-Time (JIT) mode, the transportation and completion times of PMCUs are uncertain; (2) cruise ship production is subject to strict plan constraints, and the scheme needs to match the plan as closely as possible while maintaining efficiency; (3) the time required for different equipment to transport different PMCUs is uncertain, which is influenced by several factors, such as worker proficiency, yard placement, etc.; and (4) the number of equipment resources is limited, and multiple types of jobs share the same type of equipment. Although some of the cases related to the logistics industry also have such characteristics, the application of a large number of automated equipment makes the logistics process more efficient, accurate, and collaborative. The transportation time and delivery time can be effectively described by using fixed time.

At present, existing studies on ship intermediate product logistics [12,13,14,15] have not considered the characteristics of cruise ship PMCU logistics comprehensively. During actual PMCU logistics, the domestic cruise manufacturer mainly adopts human decision making and on-site scheduling, lacking the scheduling methodology oriented to the overall cruise project, which leads to the independence of the various parts of the process, resulting in low efficiency and accuracy. Under these circumstances, the main contributions of this study can be summarized as follows:

1. This paper formulates a multi-objective fuzzy equipment collaborative scheduling model for PMCU logistics, predicated on maximizing the average agreement index of fuzzy due dates and minimizing the maximum fuzzy makespan, thereby addressing inherent uncertainties in cruise shipyard operations.
2. A new genetic hyper-heuristic algorithm with a self-learning mechanism (GA-SLHH) is proposed in this paper. In the GA-SLHH framework, low-level heuristics (LLHs) are composed of a set of classical scheduling rules. The high-level strategy (HLS) is composed of a genetic algorithm (GA) and self-learning mechanism. The HLS iteratively optimizes the LLHs, which, in turn, control the solution of the collaborative scheduling model of PMCU logistics.
3. The feasibility and applicability of the GA-SLHH proposed in this paper are verified through numerical experiments and enterprise case verification with well-known meta-heuristic algorithms (i.e., GA and PSO), classical scheduling rules, and genetic hyper-heuristic algorithms without a self-learning mechanism (GA-HH).

The remainder of the paper is organized as follows: Section 2 gives a review of the related literature. Section 3 presents the problem description and proposes a mixed-integer linear programming formulation. Section 4 proposes a self-learning hyper-heuristic algorithm based on a genetic algorithm (GA-SLHH). The design of the numerical experiments and analysis of the results are given in Section 5. Section 6 suggests approaches of research for enterprise decision makers and management. Section 7 gives the conclusions of this paper and directions for future research.

## 2. Related Research

Scheduling in the shipbuilding industry involves the strategic allocation and organization of resources, personnel, and equipment, in accordance with work plans and real-world conditions. To facilitate coordinated operations and achieve predefined objectives. Existing research in the shipbuilding industry has focused on specific scheduling areas including workshop scheduling [16,17,18,19,20,21], transport scheduling [12,22,23,24,25], spatial scheduling [26,27,28], project scheduling [29,30,31], yard scheduling [32,33], etc. Nonetheless, these studies largely address isolated shipbuilding processes. The complex challenges posed by collaborative scheduling, which necessitates the integration of multiple operations and diverse equipment types, remain underexplored. This type of collaborative scheduling problem is more commonly studied in the logistics industry, represented by container terminals [34,35]. Related studies tend to be orientated toward logistics scenarios with a high prevalence of automated equipment, while the studies are also characterized by unidirectionality and homogeneity. The scheduling problem within the cruise ship manufacturing enterprise needs to be combined with the production operation mode to consider labor, equipment, schedule, plan agreement, and other factors, and to make a scheduling scheme under the time uncertain environment, which is more complicated compared with the logistics industry. For such problems, scholars often use fuzzy time for uncertain environment descriptions [36,37,38], converting conventional scheduling schemes into fuzzy scheduling schemes to guide schemes with more application possibilities. At the same time, in a complex decision-making process, decision-makers in a cruise ship manufacturing enterprise will develop scheduling schemes by making decisions at each decision point based on experience. Furthermore, the potential dependencies between consecutive decisions, when considered in scheduling, can offer comprehensive decision-making paradigms for the enterprise, surpassing the support of a singular scheme for isolated issues.

Research on methods for addressing similar scheduling problems includes methods such as exact algorithms [39,40], heuristic algorithms [41], and meta-heuristic algorithms [42,43]. Additionally, advancements in artificial intelligence have facilitated innovative applications in this field. For example, Serrano-Ruiz et al. [44] constructed the scheduling of an intelligent manufacturing system as a Markov Decision Process (MDP), incorporating multi-objective optimization features, and used deep reinforcement learning (DRL) to solve the job shop smart manufacturing scheduling problem. Zhang et al. [45] proposed a deep reinforcement learning method based on a scheduling network tailored to the multi-AGV scheduling environment, effectively solved the integrated scheduling problem in automated container terminals. However, these studies are not readily applicable to the cruise ship manufacturing context, which demands extensive human decision-making and manual labor, influenced by many factors.

In recent years, hyper-heuristic algorithms have garnered scholarly attention and have been successfully applied to NP-hard problems, including the job shop scheduling problem (JSP) [46], vehicle routing problem (VRP) [7], and nurse rostering problem (NRP) [47]. These algorithms effectively mitigate the impact of complex constraints on problem-solving by searching the domain of low-level heuristic (LLH) rules when solving complex NP-hard problems [48,49]. This approach offers distinct advantages in solving scheduling problems with complex constraints. For instance, Guo et al. [50] focused on reconfigurable manufacturing systems, introduced a new dynamic flexible job shop scheduling problem (DFJSP-RMC) and propose an improved genetic programming hyper-heuristic (GP-HH) method to solve this problem. Zhang et al. [51] constructed a multi-objective fuzzy multitask-oriented manufacturing service composition model considering urgent task arrivals and delayed delivery times, aiming to optimize the manufacturing service combination problem in cloud manufacturing environments, and designed a genetic hyper-heuristic algorithm with variable chromosome lengths to achieve efficient solutions. Song and Lin [52] used a tree structure to guide heuristic sequence coding for the distributed assembly permutation flow-shop scheduling problem, which features a sequence-dependent setup. Lim et al. [53] used simulated annealing (SA) as a framework to design hyper-heuristic algorithms with machine assignment rules (MARs) and job-sequencing rules (JSRs) to solve the flexible job shop scheduling problem.

Some other hyper-heuristic algorithms used in the literature are summarized in Table 1. A review of these contributions reveals that most studies are limited to specific aspects and do not effectively address the cruise PMCU logistics scheduling problem. In this paper, a fuzzy multi-objective model is constructed for the first time, to solve the collaborative scheduling of yard operations and transport equipment in PMCU logistics. This model integrates two optimization objectives: minimizing the maximum fuzzy makespan and the agreement index of due date. Furthermore, it accommodates complex constraints, including bi-directional operations, multi-type tasks, and fuzzy transport times, which are inherent in PMCU logistics. As a novelty, we propose a self-learning genetic hyper-heuristic algorithm (GA-SLHH) in this paper to solve the scheduling problem. The proposed algorithm employs dispatching rules as components of low-level heuristics (LLHs) and employs a genetic algorithm as a high-level strategy (HLS) to optimize LLHs through selection, crossover, mutation, and iterative processes. Additionally, it introduces probabilistic self-learning models to sufficiently explore the relevance of dispatching rules. Finally, the validity of the method is demonstrated through example validation.

## 3. PMCU Logistics Collaborative Scheduling Problem

### 3.1. Problem Description

**Figure 2:**
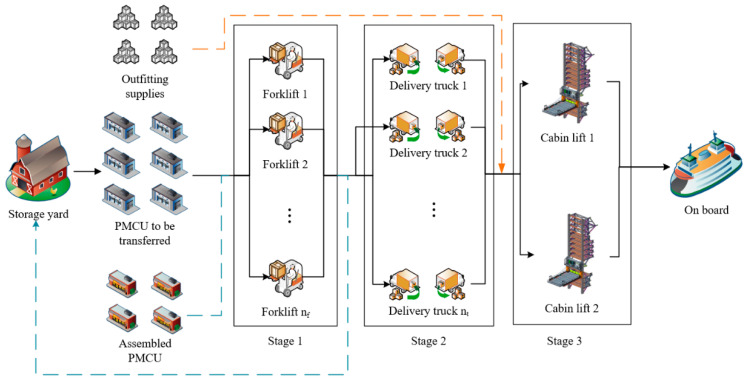
PMCU logistics collaborative scheduling problem.

The structure of the PMCU logistics collaborative scheduling problem studied in this paper is illustrated in Figure 2. The logistics process involves multiple stages of transportation, including assembled PMCU, PMCU awaiting transport, and outfitting supplies requiring forklifts, delivery trucks, and cabin-lift, respectively. Among these, the PMCUs awaiting transport requires the most diverse type of equipment. Initially, they are first removed from the yard using forklifts and then placed onto delivery trucks, which transport the PMCUs to the dock, where they are embarked by cabin lift. The assembled PMCU is a cabin that has just completed the final stage of assembly on the assembly line when it needs to be removed by a forklift and positioned appropriately in the PMCU yard. Due to the large number and weight of outfitting supplies, cabin lifts are usually used for embarkation in cruise ship production. Therefore, it is also necessary to consider the occupation of the cabin lift by outfitting supplies during the final stage of logistics.

The collaborative scheduling problem of PMCU logistics can be described as follows: There are N different types of transportation tasks; for different transportation tasks i, the same or different number of transfer stages s need to be transported. There are n equipment k in each stage, and the processing time of different equipment k for the same task i is different. Each task can only be transported on transport equipment, each transport equipment can only handle one transportation task at a time. This problem can be classified as a collaborative scheduling problem with bi-directional and multi-type task constraints, making it more complex than typical collaborative scheduling problems.

### 3.2. Environmental Conditions and Constraints

#### 3.2.1. Uncertain Environment

**Figure 3:**
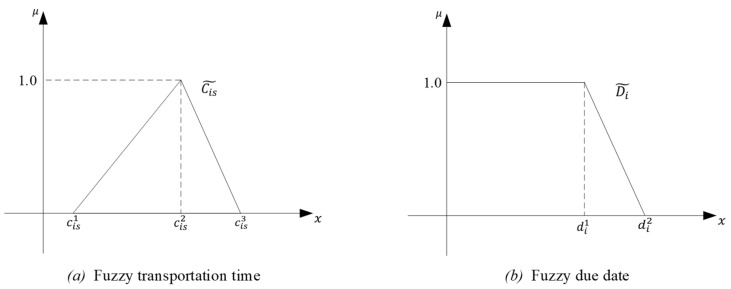
Fuzzy time.

The production and construction of ships fall within the large-scale equipment manufacturing industry. Compared with the industries that use a large number of automotive equipment, the adoption of automated and standardized production is relatively limited. Consequently, many production tasks need to be completed manually, resulting in significant uncertainty regarding the processing times of various tasks. As a typical link in cruise production, PMCU logistics also exhibits the characteristics of uncertain transportation time and due date. This kind of task scheduling process with uncertain time is usually described by fuzzy time. The transportation time is described by triangular fuzzy numbers (TFNs), as shown in Figure 3. 

In Figure 3, the vertical axis μ represents the membership function of fuzzy time, while the horizontal axis represents fuzzy time. In Figure 3a, where $c_{is}^{1},c_{is}^{2},c_{is}^{3}$ denote the earliest, expected, and latest completion times of the transport task i in stage s, respectively. Regarding due dates, only the latest demand time is usually constrained in actual manufacturing. Therefore the fuzzy time description for the due date in [61] is adopted, as illustrated in Figure 3b.

When solving the scheduling problem, it involves the operations of TFNs. For two TFNs $\tilde{C_{1}}=(c_{1}^{1},c_{1}^{2},c_{1}^{3})$ and $\tilde{C_{2}}=(c_{2}^{1},c_{2}^{2},c_{2}^{3})$, the calculation and ranking criteria of fuzzy numbers are based on the approaches in [61], as shown in Table 2.

For the two TFNs $\tilde{C_{1}}$ and $\tilde{C_{2}}$, the +(sum) operation is the addition of the earliest, expected, and latest time, respectively. The ⋁(max) operation takes the largest values of the earliest, expected, and latest time of the two TFNs, respectively. The ranking operation is a sequential comparison process. For the TFNs $\tilde{C_{1}}$ and $\tilde{C_{2}}$, first compare $C_{1}(\tilde{C_{1}})$ and $C_{1}(\tilde{C_{2}})$. If $C_{1}(\tilde{C_{1}})$>$C_{1}(\tilde{C_{2}})$, then $\tilde{C_{1}}>\tilde{C_{2}}$. If $C_{1}\left(\tilde{C_{1}}\right)<C_{1}(\tilde{C_{2}})$, then $\tilde{C_{1}}<\tilde{C_{2}}$. If $C_{1}\left(\tilde{C_{1}}\right)=C_{1}\left(\tilde{C_{2}}\right),$ then we need to further compare the values of $C_{2}(\tilde{C_{1}})$ and $C_{2}(\tilde{C_{2}})$. Similarly, if $C_{2}\left(\tilde{C_{1}}\right)=C_{2}(\tilde{C_{2}})$, then we need to further compare the values of $C_{3}(\tilde{C_{1}})$ and $C_{3}(\tilde{C_{2}})$.

#### 3.2.2. Transport Equipment Constraints

Equipment constraints in a scheduling problem are typically defined based on the specificities of the actual problem environment. Within the PMCU logistics collaborative scheduling problem, worker proficiency in operating transport vehicles and placing cabins introduces variability in transportation times for different PMCUs by various types of equipment. This also leads to the fact that the selection of transport equipment at different stages of the PMCU will have a great impact on the final scheduling scheme. Compared to traditional scheduling problems, the solution space and optimization of this problem are considerably more complex.

In addition, during the final stage of logistics, due to the impact of the deck layout established in the early stage of design, the target locations of different prefabricated cabins are greatly different from the distance to different cabin lifts. Therefore, in the actual environment, the designated cabin lift for embarking the cabins will be fixed and restricted to ensure the smooth subsequent on-board installation. Therefore, when solving the scheduling problem presented in this paper, it is necessary to consider the constraints of specific task-processing equipment to ensure that the scheduling scheme meets the actual requirements, and also puts forward higher requirements for algorithm design and constraint processing.

### 3.3. Assumptions

Combined with the actual environment of logistics of cruise ship production, the assumptions of PMCU logistics collaborative scheduling studied in this paper are shown in Table 3.

### 3.4. Mathematical Model

The relevant definitions and descriptions of symbols in the collaborative scheduling model of PMCU logistics are shown in Table 4.

**Figure 4:**
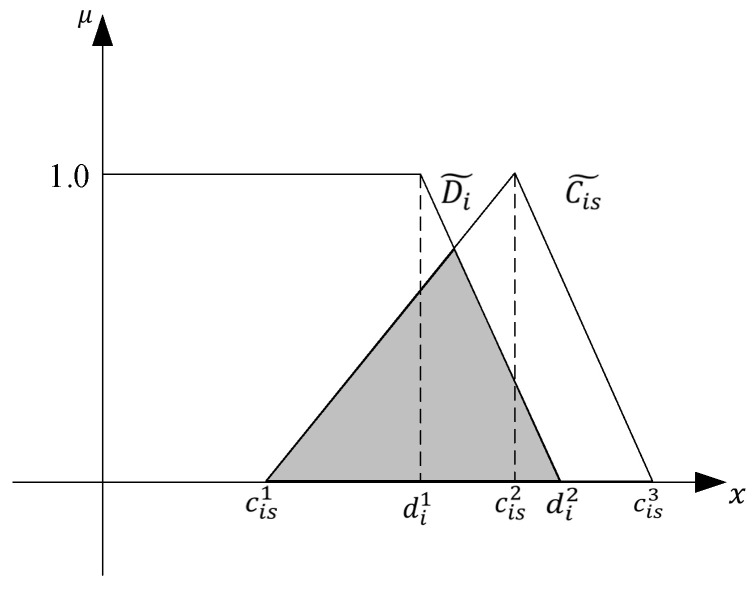
Agreement Index.

For cruise ship production processes characterized by complex constraints, manufacturers often require not only efficient production but also execution as closely as possible to the established plan. Therefore, for the collaborative scheduling problem of PMCU logistics studied in this paper, the optimization objectives should include minimizing the maximum fuzzy makespan and maximizing the average “Agreement Index” (AI) for each task relative to the planned due date. Specifically, these objectives are as follow:

(1) $$min\;f_{1}=\tilde{C_{max}}=\max\tilde{C_{i}}$$

(2) $$max\;f_{2}=AI=\frac{1}{n}\sum_{i=1}^{n}AI_{i}$$

where $AI_{i}$ represents the agreement index of completion time and planned due date of task i, as shown in Figure 4.

Then $AI_{i}$ is calculated as follows:

(3) $$AI_{i}=\frac{area(\tilde{C_{i}}\cap \tilde{D_{i}})}{area(\tilde{C_{i}})}$$

To facilitate optimization and reduce the amount of computation, and to facilitate the scheduler to generate corresponding scheduling schemes based on varying conditions, we assign different weights to the two objective functions and combine them into a single objective function:

(4) $$F=\omega_{1}\cdot f_{1}+\omega_{2}\cdot {1/f}_{2}$$

(5) $$\omega_{1}+\omega_{2}=1$$

In order to avoid the impact on the objective function due to the inconsistency of the two optimization objectives changing drastically, the optimization objective $f_{1}$ is normalized according to Equation (6).

(6) $$N\left(f_{1}\right)=\frac{f_{1}-{\left(f_{1}\right)}_{min}}{{\left(f_{1}\right)}_{max}-{\left(f_{1}\right)}_{min}}$$

In Equation (6), ${\left(f_{1}\right)}_{max}$ and ${\left(f_{1}\right)}_{min}$ represent the upper and lower bounds of the objective $f_{1}$. And the objective function is shown as Equation (7).

(7) $$F=\omega_{1}\cdot N\left(f_{1}\right)+\omega_{2}\cdot \left({1/f}_{2}\right)$$

The constraints of the problem model include:

(8) $$\tilde{C_{is}}=\left(1-Y_{is}\right)\cdot \tilde{C_{is-1}}+Y_{is}\cdot (\tilde{B_{is}}+\sum_{k=1}^{n_{m}}X_{iks}\cdot \tilde{P_{iks}})$$

(9) $$\tilde{C_{is}}\leq \tilde{B_{is+1}}$$

(10) $$\tilde{B_{i1}}\geq \tilde{R_{i}}$$

(11) $$\sum_{k=1}^{n_{m}}X_{iks}=1$$

(12) $$\tilde{B_{is}}\geq (\tilde{C_{js}}-Z_{ijks}\cdot M)\cdot Y_{is}\cdot Y_{js}$$

(13) $$\tilde{C_{is}},\tilde{B_{is}}\geq 0$$

Equation (8) defines the completion time of each task at each stage, and, if task i does not go through the transportation stage s, then its completion time of task i at that stage is equal to the completion time of the previous stage that it went through. Equation (9) ensures that each task i must complete the previous before it can start the next stage. Equation (10) represents that task i is released for transportation. Equation (11) indicates that each task can only be processed on one transport equipment at the same time. Equations (12) and (13) describe the priority constraints of tasks on the same equipment in the same stage. Equation (12) indicates that the start time of the equipment k of task i in stage s must be after the completion time of task j. Equation (13) represents the fuzzy makespan and the fuzzy beginning time of task i must be greater than zero.

## 4. Self-Learning Hyper-Heuristic Algorithm Based on Genetic Algorithm

### 4.1. Hyper-Heuristic Algorithm Based on Genetic Algorithm (GA-HH)

**Figure 5:**
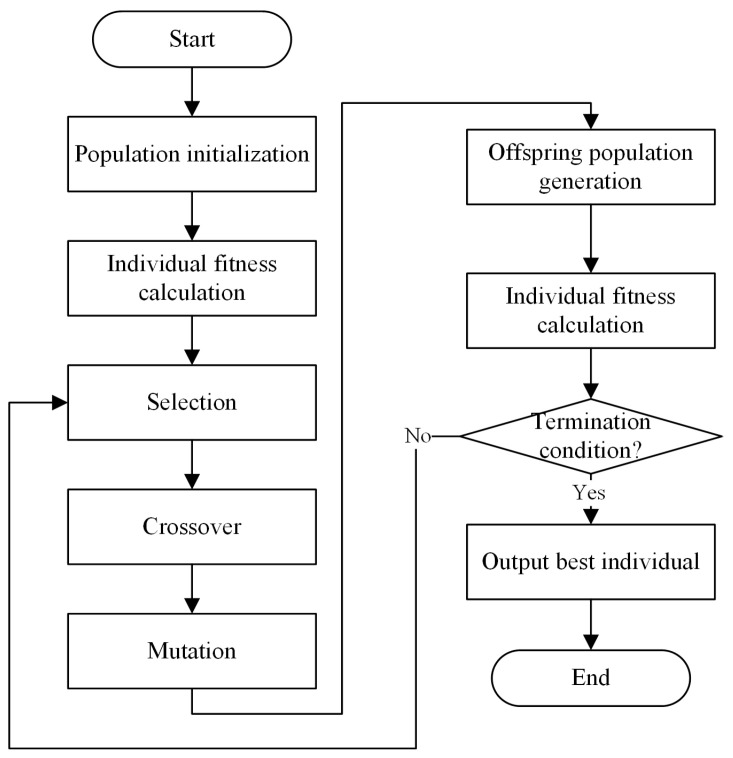
Flowchart of genetic algorithm.

The genetic algorithm (GA) is an algorithm based on the principles of evolutionary theory, first proposed by American scholar John Holland in 1975 [62]. Its basic idea is to find a high-quality feasible solution by modeling the process of biological evolution observed in nature. The core of the genetic algorithm begins with an initial population and uses genetic operations to generate new populations iteratively to search for an acceptable solution suitable for the specific problem at hand. The GA usually includes population initialization, selection, crossover, and mutation operations. The execution flowchart of genetic algorithm is shown in Figure 5.

In most studies, genetic algorithms are used as a tool for the solution, usually directly for generating combinatorial solutions in large batches, and then improving convergence accuracy and speed by enhancing the quality of the initial population or improving the genetic manipulation methods. However, in the complex environment of real scheduling problems, genetic operations usually produce a significant amount of infeasible solutions, requiring a large number of solution judgments and repair operations on the offspring population, which consumes a large amount of time and cost. Compared with traditional genetic algorithms, hyper-heuristics do not directly change the combinatorial scheme but manipulates the set of LLHs through genetic operations to change the combinatorial scheme, which has a higher flexibility and applicability to complex problems. Consequently, the positions of the superior individuals in the population do not represent the direction of optimization. It is necessary to avoid the mutual influence of individuals within the same generation population, which will cause disturbance to the iteration optimization. Compared with other meta-heuristics, the GA can directly pass the better individual information of the parent to the offspring, which is more suitable as HLS.

In this paper, the genetic algorithm is not directly used to generate and manipulate the scheduling scheme, but as an HLS used to control the LLHs. A new set of LLHs is generated by genetic operation iteratively, and then the scheduling scheme is subsequently calculated based on the LLHs. Finally, the best-fit feasible LLH that meets the current problem is generated to achieve the high-quality feasible solution for the scheduling problem.

### 4.2. Low-Level Heuristics

In hyper-heuristic algorithms, the search space of HLS is composed of LLHs that conform to the constraints of the problem. In some of the hyper-heuristic algorithm studies, the LLH is a neighborhood operator such as swap and insert, which increases the diversity of the solution space through neighborhood changes to obtain a higher-quality solution [46,51,63]. It is still essentially a scheduling scheme that solves the problem directly.

Unlike the neighborhood operator, the genetic hyper-heuristic algorithm proposed in this paper will use a combination of 14 dispatching rules that match the specific characteristics of the problem to construct the LLH, transforming the search space of the algorithm into a rule space in which genetic operations are used to find the best-fit rules. Dispatching rules are used as LLHs because of their successful application in scheduling problems, on the one hand, and also because they are more suitable for manual decision-making in actual environments. In addition, dispatching rules as LLHs have better extensibility and flexibility, and the corresponding rules can be added or deleted according to the actual conditions without affecting the structure of the HLS structure. The 14 dispatching rules used in this paper are shown in Table 5.

Typically, a comprehensive scheduling solution for a scheduling problem can be found directly by using only one dispatching rule, but the result of using the same dispatching rule for all decision points in a scheduling problem may not be optimal. The use of a combination of multiple dispatching rules has been shown to provide a better solution compared to using a single rule [65], and we will obtain a sequence of best-fit feasible hybrid dispatching rules that satisfy the problem through continuous iterations of a high-level genetic algorithm.

After determining the composition of the LLHs, it is then necessary to specify how the scheduling scheme can be solved from the LLHs. In dispatching rule-based scheduling, the equipment corresponding to each task at different stages of the whole process is usually determined based on a certain scheduling rule to form a complete scheduling scheme. For the problem studied in this paper, a complete scheduling program should cover all decision points, including all tasks of PMCUs and outfitting supplies, as well as the equipment arrangement for each task at each stage. The solving process of the scheduling scheme used in this paper is shown in Table 6.

### 4.3. High-Level Strategy

In this paper, we employ the genetic algorithm as a high-level heuristic strategy, which is the same as the traditional genetic algorithm framework. This framework comprises four parts: population initialization, selection, crossover, and mutation.

In addition to the basic genetic operations, the encoding and decoding mechanisms need to be designed. In genetic algorithms, the encoding and decoding mechanisms usually need to be designed to combine with the specific problem to be solved, while, in the GA-SLHH, the encoding and decoding mechanisms also have the function of information transfer between LLHs and HLS. Therefore, the design of encoding and decoding mechanisms needs to fully consider the problem characteristics and the solution process.

1. Encoding and decoding

In the GA-HH, the encoding will be represented as a floating-point number between 0 and 1. A chromosome represents an LLH containing multiple hybrid dispatching rules. And concerning the way the float numbers are mapped to dispatching rules, we use a roulette wheel approach; i.e., each dispatch rule initially has the same probability of taking a value—for the value corresponding to a gene position on a chromosome, we obtain the integer encoding of the dispatching rule corresponding to that floating point number by roulette and then generate an LLH to realize the decoding. 

**Figure 6:**
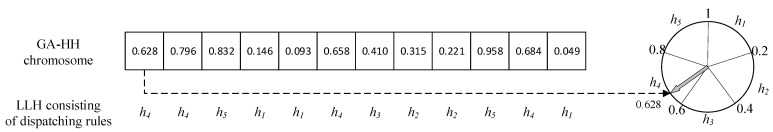
Chromosome and dispatching rules mapping.

The encoding and decoding are illustrated using a problem with four tasks to be transported, each requiring three stages as an example. For this problem, five dispatching rules can be used to generate LLHs, and, in the initial state, all five dispatching rules are taken with an equal probability of 0.2. In high-level genetic algorithm operations, the structure of a chromosome and the LLH it maps to are shown in Figure 6. In the GA-SLHH chromosomes, a floating-point number at a locus represents a mapping probability, and the interval in which this probability lies determines the dispatching rule. When the chromosome is decoded, LLHs consisting of dispatching rules for each decision point are also generated. It can be seen that the GA-SLHH does not directly search the scheduling scheme solution space but optimizes the scheduling scheme by optimizing the dispatching rule for executing the scheduling, and does not need to directly solve the complex scheduling problem.

We used 14 dispatching rules as components for generating the LLHs, with each heuristic rule having the same initial probability distribution, thus ensuring that the probability of choosing each of the 14 scheduling rules is the same at each decision point in the HLS. The length of each chromosome length is designed to ensure that the number of generated dispatching rules is equal to the number of decision points to ensure that all tasks have a dispatching rule that can satisfy the appropriate assignment of them to the transportation equipment of the corresponding stage for processing at the stage they need to go through.

2. Population initialization

A complete chromosome should cover all decision points in the scheduling problem and decode the heuristic rules to generate a comprehensive scheduling solution by realizing the probability of roulette. In order to ensure that all dispatching rules can be selected with equal probability, we use a random population initialization strategy that obeys a 0–1 uniform distribution to randomly generate a sequence of random numbers that meets the number of decision points in the problem, forming a chromosome to generate the initial population.

3. Selection

In genetic algorithms, solutions with higher fitness are more likely to be used for selection. Therefore, roulette wheel selection is used at this stage to measure the probability that an individual will make it to the next generation based on its fitness in the population. The probability of roulette selection is calculated as shown in Equation (14), where $fit_{s}$ denotes the fitness of the current individual.

(14) $$P(s)=\frac{fit_{s}}{\sum_{i=1}^{n}fit_{i}}$$

4. Crossover

**Figure 7:**
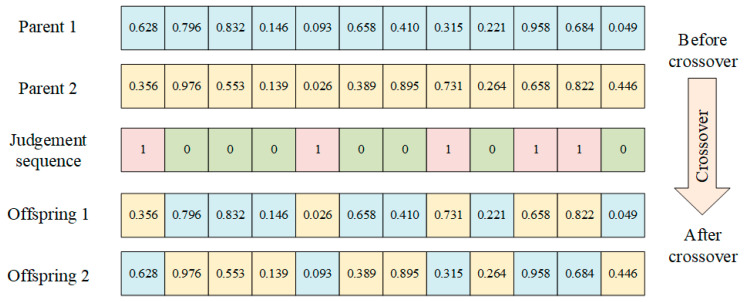
Uniform crossover operator.

Genetic algorithms maintain population diversity through crossover operators, which usually need to be designed for different problems. The general crossover operator in the basic genetic algorithm generates two or more long subsequences in the offspring individuals. In the hyper-heuristic proposed in this paper, the HLS operates directly on a set of LLHs rather than on the solution space of the scheme. Consequently, different dispatching rules may lead to the same scheme in the decoding computation, leading to a lack of population diversity. The uniform crossover operator can reduce the length of offspring individual subsequences, increase the number of subsequences, and improve the diversity of offspring population. Thus, we used the uniform crossover to ensure that we maximize diversity in the LLH generation scheduling scheme within the population. During the crossover operation, we generate a 0–1 sequence of the same length as the chromosome. If the value on the corresponding position p is 0, the parent does not perform crossover at position p, and, conversely, if the value is 1, the parent performs crossover. The uniform crossover operator is shown in Figure 7.

5. Mutation

**Figure 8:**
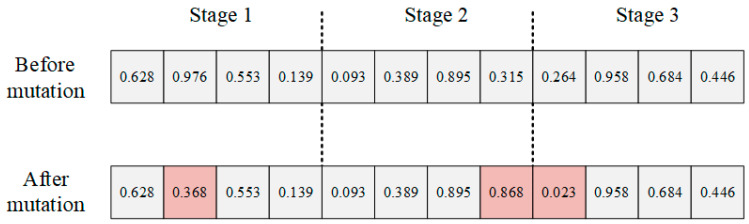
Mutation operator.

Similar to the crossover operator, mutation is performed only on the LLH sequence made up of the scheduling rules, without taking into account the relevant constraints present in the actual scheduling environment. Since the PMCU logistics process includes three stages, providing perturbation for each stage can effectively improve the exploration and exploitation capability of the algorithm compared to the conventional mutation operator. In this study, the mutation operator in the HLS will add perturbations at each stage when executing the decision, so a multi-point mutation, split by stage, will be used, as shown in Figure 8.

6. Iteration

The LLH sequences computed by HLS, along with selection, crossover, and mutation operators, will carry information about the current problem solution of the parent to the next-generation population. This information is subsequently decoded and mapped to generate a new complete scheduling scheme. Repeat the above process and iterate the computation until the termination condition is reached to output the feasible solution. In order to improve the convergence speed, the LLHs that do not enhance the population fitness will be ignored when iterating for each generation of the population. In addition, in order to explore the potential relevance between decisions, a self-learning mechanism will be introduced at each generation of the population iteration, and the relevant introduction about the self-learning mechanism will be provided in Section 4.4.

### 4.4. Self-Learning Mechanism

In this paper, when HLS operates on LLH, multiple dispatching rules may correspond to the same scheduling action. The GA mainly maintains population diversity through the crossover operator; however, the individual gene values remain unchanged, resulting in the corresponding dispatching rule is unchanged. Simply increasing the mutation probability or the number of mutated genes does not effectively solve this problem. It is necessary to design an adjustment scheme for mapping probability based on the specific problem.

In actual PMCU logistics equipment scheduling, it is often the case that the decision made by the decision-maker at a previous decision point has a potential impact on succeeding decisions and even on the final scheme. Reflected in the computation is also rthe potential relevance of adjacent dispatching rules for the generation of the problem solution. Therefore, we propose that the mapping probability of a dispatching rule in LLHs is variable, and the effects of component adjacencies needs to be considered when determining the probability of a certain dispatching rule takes a value at a certain position. The use of genetic algorithms in HLS alone cannot explore the potential influence between decision actions, nor can it completely and accurately evaluate whether the generated scheduling scheme is entirely applicable to the current problem environment. At this point, relevant knowledge can be obtained from scheduling solutions that have already been identified in previous evaluations as more suitable for the current problem environment, and the algorithm learns the knowledge to adjust the subsequent search scope and optimization direction to provide decision-making paradigm support for the enterprise.

For the statistical analysis and computation of knowledge, we use the knowledge and its statistics derived from the probabilistic learning model presented in the study by Shao et al. [66], as shown in Equation (15).

(15) $$\mu_{h,q}=\frac{1}{2}(\frac{\rho_{h,q}}{\sum_{r\in \Omega_{q}}\rho_{r,q}}+\frac{\eta_{h^{\prime },h}}{\sum_{r\in \Omega_{q}}\eta_{h^{\prime },r}})$$

In Equation (15), $\rho_{h,q}$ represents the number of times the h-th dispatching rule occurs before (and including) position q in the current population. $\eta_{h^{\prime },h}$ represents the number of times the h-th dispatching rule component occurs immediately after the $h^{\prime }$-th dispatching rule in the current population. $\mu_{h,q}\;$represents the probability of selecting the h-th scheduling rule at position q. $\Omega_{q}$ is the set of dispatching rules that can be assigned at position q. In the GA-SLHH proposed in this paper, LLH is a sequence consisting of a classical dispatching rule. The specific dispatching rule is determined by the probability of each position on the chromosome (as shown in Figure 6). The self-learning model updates the probability ranges by counting the occurrences of a dispatching occurs at position q and the neighboring dispatching rules for all individuals during the population iteration (i.e., $\mu_{h,q}$).

Knowledge is usually summarized by generalizing existing information. We considered that, in the iterative optimization process of hyper-heuristic algorithms, knowledge consists of two parts: explorative knowledge and accumulative knowledge. Explorative knowledge refers to the knowledge obtained from the superior individuals in the current population, while accumulative knowledge refers to the knowledge obtained from the set of historically best-fit individuals as the iterative process proceeds, and the combination of these two types of knowledge guide the algorithm’s subsequent optimization direction together. We designed a self-learning mechanism to guide the hyper-heuristic algorithm, as shown from Equations (16)–(18).

(16) $$\mu_{h,q}=\frac{Iter}{maxIter}\mu_{h,q_{his}}+\frac{maxIter-Iter}{maxIter}\mu_{h,q_{cur}}$$

(17) $$\mu_{h,q_{his}}=\frac{1}{2}(\frac{\rho_{h,q_{his}}}{\sum_{r\in \Omega_{q}}\rho_{r,q_{his}}}+\frac{\eta_{h^{\prime },h_{his}}}{\sum_{r\in \Omega_{q}}\eta_{h^{\prime },r_{his}}})$$

(18) $$\mu_{h,q_{cur}}=\frac{1}{2}(\frac{\rho_{h,q_{cur}}}{\sum_{r\in \Omega_{q}}\rho_{r,q_{cur}}}+\frac{\eta_{h^{\prime },h_{cur}}}{\sum_{r\in \Omega_{q}}\eta_{h^{\prime },r_{cur}}})$$

In Equation (16), $\mu_{h,q_{his}}$ represents the accumulative knowledge, which is computed from the set consisting of historically best-fit individuals iteratively generated by the algorithm. This reflects the probability that the h-th dispatching rule occurs at position q from the historically best-fit population. And $\mu_{h,q_{cur}}$ represents the exploratory knowledge, computed from the summarization of the better individuals in the current population, which is the probability that the h-th dispatching rule occurs at position q from the current population. maxIter and Iter denote the maximum number of iterations and the current number of iterations of the algorithm, respectively. It means that, at the beginning of the hyper-heuristic algorithm, without accumulating enough historical excellent experience to learn, the algorithm will tend to learn from the acquired exploratory knowledge, and, with the accumulation of knowledge and experience progressively, the algorithm will tend to learn more and more from the accumulated knowledge. This is also consistent with the tendency of enterprise managers to make production schemes regarding experience.

### 4.5. GA-SLHH Flow

We believe that self-learning mechanisms should be integrated into the iterative process of HLS to serve as a guide for the search direction of the algorithm. At the same time, the genetic algorithm should also retain its “survival of the fittest” feature to ensure that each iteration explores the optimization direction with a certain probability.

Therefore, we decided to use both approaches to collaboratively drive the algorithm iterations, with each approach generating half the number of LLH individuals in the new population. The main difference is that the individuals selected through the genetic algorithm are converted to LLH using roulette probabilities based on the initial state probabilities, meaning that all dispatching rules have equal probabilities. The individuals generated through the self-learning mechanism are converted to LLH using the probabilities calculated in Equations (16)–(18). 

**Figure 9:**
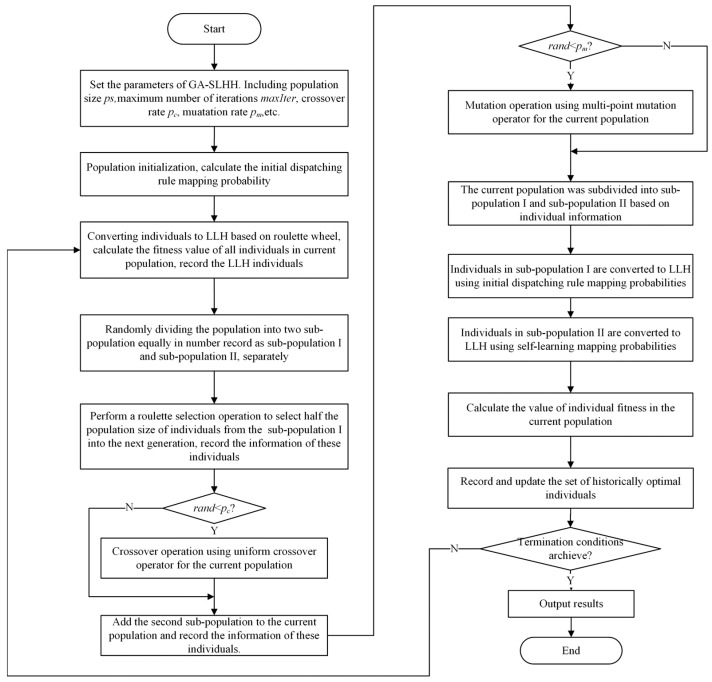
GA-SLHH flowchart.

The flowchart of the GA-SLHH is shown in Figure 9. And the pseudo-code for the GA-SLHH is provided in Algorithm 1.
**Algorithm 1:** GA-SLHH**Input:** ps$:\;\mathrm{Population}\;\mathrm{size},\;p_{c}$$:\;\mathrm{Crossover}\;\mathrm{rate},\;p_{m}$: Mutation rate, maxIter: Maximum number of iterations **Output:** High-quality feasible scheme **Begin** i=1; $\mathrm{Initialize}\;P\left(i\right)$ // Randomly generate an initial population that satisfies a (0,1) uniform distribution $P\left(i+1\right)=\emptyset$ $f=zeros\left(PS,1\right)$ while (i≤maxIter) **do**  for j=1 to ps **do**   Evaluate the fitness of population P(i); // Fitness evaluation based on Equations (1)~(7);  **endfor** forj=1 to ps **do**   Selection of individual parents based on fitness f; $\;\;\mathrm{if}\;rand<p_{c}$    Perform crossover operation to generate offspring P(i+1) individuals;   **else** $\;\;\;\mathrm{Directly}\;\mathrm{generate}\;\mathrm{offspring}\;P\left(i+1\right)\;$individuals;   **endif** **endfor**  for j=1 to ps **do** $\;\;\mathrm{if}\;rand<p_{m}$    Perform multi-point mutation operation to individuals in P(i+1);   **endif** **endfor**  Divide the population P(i+1) into two sub-populations sub-population I and sub-population II with the same number of individuals;  for j=1 to ps/2 **do**
  Convert individuals in sub-population I to LLHs using initial mapping probabilities;   Convert individuals in sub-population II to LLHs using self-learning mapping probabilities; **endfor**  Fitness evaluation of population P(i+1); $\;P\left(i\right)=P(i+1)$;  i=i+1;  **endwhile** **end**

## 5. Computational Experiments

To assess the applicability and performance of the algorithm proposed in this study, we designed two groups of numerical experiments. All experimental verification programs were executed on a computer equipped with an AMD Ryzen 5 4600H 3.00 GHz processor and 16 G of running memory.

(1) Performance testing. The GA-SLHH, along with the commonly used meta-heuristic algorithms GA and PSO, and the commonly used scheduling rules FIFO, SPT, MWKR, and FRO are used to perform computations under the same instances of fuzzy scheduling problems, respectively, which, in turn, evaluates the performance of the GA-SLHH. In addition, the GA-HH algorithm without a self-learning mechanism will also be used as one of comparison approaches to evaluate the effectiveness of the self-learning mechanism designed in this study.

(2) Case verification. The practicality of the GA-SLHH in this study is assessed through the enterprise case of PMCU logistics equipment operation of a cruise ship manufacturer for collaborative scheduling.

### 5.1. Performance Testing

To evaluate the performance of the proposed algorithm in this study, experiments will be performed using benchmark instances from the research presented in [67] concerning the fuzzy scheduling problem. Each instance included 10 tasks and 10 stages of operation per task, and the task processing time can be seen as a triangular fuzzy number. Additionally, to maintain consistency with the mathematical model proposed in this study, the due date for all tasks is established according to the method outlined in [61]. The expected processing time (e.g., $c_{i}^{2}$) for task i at each stage is summed and multiplied by a random number of [1.5,1.75] as $d_{i}^{1}$ of the due date of task i. Then, each $d_{i}^{2}$ is determined by adding a randomly generated number on the closed interval [3,15] to each $d_{i}^{1}$.

The relevant parameter settings required for the meta-heuristic and hyper-heuristic algorithms are shown in Table 7. All algorithms use the same population size and maximum number of iterations: the population size ps=30 and maximum iteration maxIter=150. The maximum CPU time for different algorithms is set to 180 s. In particular, for the GA, the chromosome representation is based on integer encoding consisting of transportation stage decision points, which is similar to the GA-SLHH. Each chromosome represents the order in which tasks are processed at each stage. Population initialization is using random generation. The crossover operator is single-point crossover, which is the same as the general GA. The mutation operator is single-point mutation, which is the same as the general GA.

In order to avoid the influence of random factors of algorithms on the results generated by the scheduling scheme, we will run each algorithm thirty times for each benchmark instance. Then, calculate the average value of the objective function $F_{avg}$, the maximum fuzzy makespan $\;f_{1}$, and the delivery date consistency index $f_{2}$. Meanwhile, in order to evaluate the optimization ability of the multi-objective method, the weights of the two optimization objectives in the objective function for the experiment are set as $\omega_{1}=0.5,\omega_{2}=0.5$, and the experimental results are shown in Table 8.

In all instances, the GA-SLHH proposed in this study outperforms other methods regarding the optimization of the objective function $F_{avg}$, and the optimization improvements compared to the GA, PSO, GA-HH, FIFO, SPT, MWKR, and FRO is, on average, 48.85%, 49.01%, 16.75%, 20.42%, 17.21%, 67.17%, and 28.23%, respectively. Under the current weight settings, the average optimization effect of the GA-SLHH is slightly worse than that of the SPT rule for the optimization objective $f_{1}$ only in Instance 1, with a difference of approximately 4.2%. This discrepancy is due to the tendency of the GA-SLHH to optimize towards $f_{2}$. Compared to other methods, the average optimization at $f_{1}$ is improved by, 23.38%, 23.98%, 5.76%, 6.77%, 10.31%, 22.83%, and 13.3%, respectively. In addition, compared with other methods, the GA-SLHH will trade off the optimization objective $f_{2}$ optimization effect for different problems to maintain a better $F_{avg}$ optimization effect. Compared to other methods, the average optimization effect is 45.46%, 55.54%, −11.19%, −12.7%, -15.47%, 167.57%, and 1.75%.

**Figure 10:**
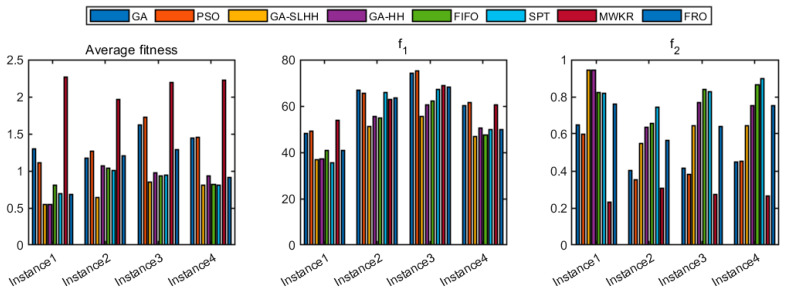
Average fitness values of 30-time calculations on benchmark instances.

As can be seen from Figure 10, the proposed GA-SLHH has a better optimization search ability and stability when compared with meta-heuristic algorithms and can find the better solution for all instances. Compared with the commonly used dispatching rules, the GA-SLHH will trade-off the optimization effect on a single optimization objective, but has a stronger comprehensive optimization ability and stability.

**Figure 11:**
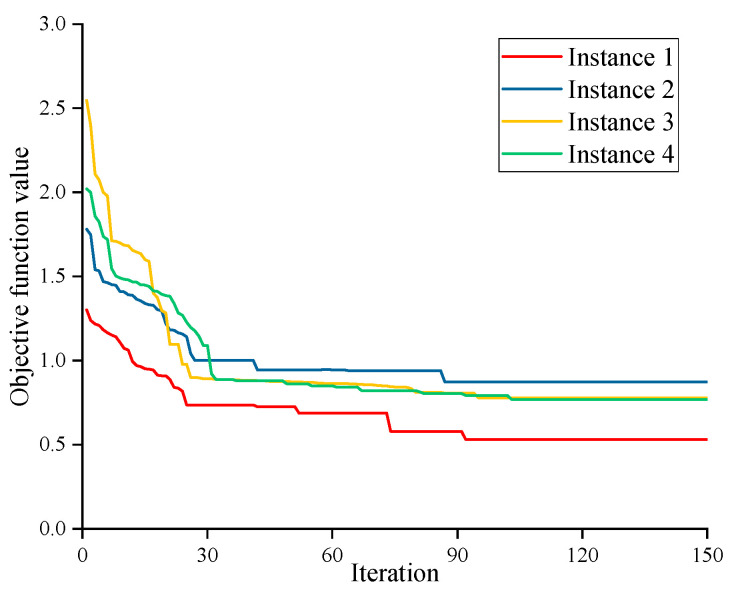
Convergence curves of GA-SLHH on benchmark instances.

Figure 11 shows the convergence curves of the GA-SLHH on each of the four benchmark instances. It can be seen that the algorithms all obtain the best value around 90 iterations and there is no large variation observed between 90 and 150 iterations. Therefore, the maximum number of iterations is set to 150 to meet the requirements of the GA-SLHH.

To further evaluate the performance of the GA-SLHH proposed in this paper for fuzzy scheduling problems, we generated multiple sets of fuzzy scheduling test cases using 15 randomly generated parameter sets. The parameters used for generating these cases are shown in Table 9. The number of operations and the processing time of each operation are randomly generated from a uniform distribution within the upper and lower limit values set in Table 9. The triangular fuzzy processing time $\tilde{C_{is}}=(c_{is}^{1},c_{is}^{2},c_{is}^{3})$ is generated as follows: Generate three random numbers $r_{1}$,$r_{2}$,$r_{3}$ in the range of process time, and then $c_{is}^{1}=r_{1}$, $c_{is}^{2}=r_{1}+r_{2}$, $c_{is}^{3}=r_{1}+r_{2}+r_{3}$, respectively. The due date is generated in the same way as in the previous part of the computational experiments.

Depending on the size of the problems, we can classify the randomly generated cases into three categories: small-scale (Cases 1–5), medium-scale (Cases 6–10), and large-scale (Cases 11–15). We continue to utilize the eight algorithms listed in Table 8 to perform performance testing on these cases. To avoid the influence of randomness, we have run the eight algorithms 30 times and recorded the value of statistical results. The experimental weight is set to $\omega_{1}=0.5,\omega_{2}=0.5$, and the mean and standard deviation (std.) values for each method are listed in Table 10.

All the best mean values in Table 10 have been bolded. It can be seen that, compared to the commonly used meta-heuristic algorithms GA and PSO, and the hyper-heuristic algorithm GA-HH, the GA-SLHH proposed in this paper has smaller mean and standard deviation values than the other methods for all sizes of problems, and exhibits a stronger capacity of optimization and stronger stability. Compared with the GA and PSO, the GA-SLHH has stronger exploration and exploitation capacities because of its hyper-heuristic hierarchical mapping decoding mechanism. At the same time, the uniform crossover operator and multi-point mutation effectively increase the population diversity during the algorithm iteration. Benefiting from the adaptive adjustment of the mapping probability through the self-learning mechanism, the GA-SLHH can be more suitable for the problem of dispatching rules, and the solution is superior to the GA-HH. Compared with dispatching rules, the GA-SLHH proposed in this paper is able to find relatively better optimization results for most of problems. It has been demonstrated that the solution of multiple dispatching rules applied together is better than that of single dispatching rule. However, in specific small-sized problems (Cases 5) and some medium-sized problems (Cases 6–7), in terms of the scheduling results obtained, the SPT outperforms the GA-SLHH, but the results obtained by the GA-SLHH are still in second place and the GA-SLHH still outperforms the other scheduling rules. Although the results obtained by the GA-SLHH may not be superior to the scheduling rules in some cases, the method is able to provide more diversity in the optimization of the scheduling scheme and satisfy the possibilities of the production decision execution. It is also important to note that, since the dispatching rule is scheduling the same problem using the same methodology at all decision points, the scheduling solutions obtained by the dispatching rules are unique, and, therefore, its result has a standard deviation value of zero.

### 5.2. Case Verification

To evaluate the applicability of the GA-SLHH proposed in this paper for the PMCU logistics collaborative scheduling problem, an instance of a production case from a cruise ship manufacturing enterprise is used for testing. The case contains eight assembled PMCUs, eight PMCUs awaiting transport, and ten batches of outfitting supplies. The transportation equipment consisting of three forklifts, four delivery trucks, and two cabin lifts. Due to factors such as the modification status of the equipment, the placement of PMCUs in the yard, and the varying heights of the target decks, the same equipment requires a different processing time for different tasks. 

In all tasks, the assembled PMCUs indicate that the assembly is complete and needs to leave the shop, requiring only one stage of the forklift operation. PMCUs awaiting transportation indicate that they need to be transported on board a ship for installation, which requires three stages of transportation: forklift operation, truck transportation, and cabin lift operation. Outfitting supplies denote materials, components, and other supplies that need to be carried on board for construction, and which need to complete only one stage of the cabin lift operation. The fuzzy operation and transportation time of the tasks on different equipment are shown in Table 11 (unit: minute). The empty values in Table 11 indicate that it does not need to be processed by the equipment at that stage.

For the two optimization objectives in this study, three sets of weights are set to represent the tendency of decision-makers in the actual scheduling. When $\omega_{1}=1$ and $\omega_{2}=0$, it means that only the fuzzy completion time is considered to be optimized during the PMCU logistics scheduling process, without considering the optimization of the average fuzzy due date agreement index. Conversely, when $\omega_{1}=0$ and $\omega_{2}=1$, it means that only the average fuzzy due date agreement index is considered to be optimized. When $\omega_{1}=\omega_{2}=0.5$, it means that two optimization objectives need to be optimized in a balanced manner. Each of the three combinations of weights represents the preferred choice of the shipyard decision-maker for PMCU logistics scheduling based on the actual project situation. The comparison method uses the methods in Section 5.1, and the relevant parameters of all the algorithms are kept the same as in Section 5.1, which are run 30 times, respectively, and the best value, the worst value, and the average value of the objective function F are counted. The case verification results are shown in Table 12.

According to the results in Table 12, it can be seen that, compared with the commonly used meta-heuristic algorithms, such as the GA, PSO, and GA-HH, which do not incorporate a self-learning mechanism, the GA-SLHH proposed in this study shows significant advantages in the cruise ship enterprise case under all three weights. In addition, due to the uniqueness of schemes based on dispatching rules, the best and worst values of the dispatching rule in Table 12 are the same as the average. Compared with the GA-SLHH, only when the weights are set to $\omega_{1}=1,\omega_{2}=0$, the scheme generated according to the rule FIFO has the same best fitness value as the GA-SLHH, and the GA-SLHH still has a significant advantage over other single dispatching rules under other weights.

**Figure 12:**
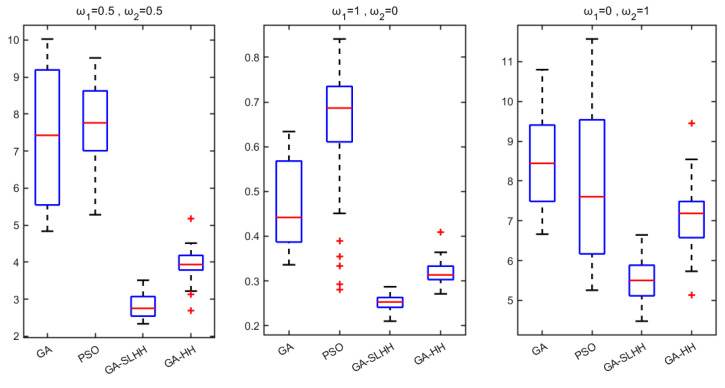
Boxplots of meta-heuristic and hyper-heuristic.

Meanwhile, to evaluate the performance differences between the meta-heuristic algorithm and the hyper-heuristic algorithm in the enterprise case, a boxplot is plotted based on the experimental results under the three sets of weight settings (i.e., Figure 12), respectively. From Figure 12, hyper-heuristics outperform meta-heuristics at different weight settings. The GA-SLHH proposed in this study outperforms other methods in terms of stability and capability of optimization search in the case of cruise ship PMCU collaborative scheduling problems. It can provide decision-makers with decision support in different directions during the production process of the enterprise.

It can be seen that the GA-SLHH proposed in this paper has a good performance in solving the PMCU logistics collaborative scheduling problem. On one hand, the improvement of the genetic operator improves the exploration and exploitation capacities of the GA-SLHH. On the other hand, the GA-SLHH utilizes the advantages of hyper-heuristic algorithms to deal with complex constrained problems, transforming the solution search space from the problem itself to the scheduling rule space, and introduces a self-learning mechanism to explore the relevance of decisions in the decision-making process, further realizing the performance improvement of the algorithm. Compared with the meta-heuristic algorithms GA and PSO, it exhibits a stronger search capability and stability under all three different weight cases. Compared with the well-known dispatching rules FIFO, SPT, MWKR, and FRO, it also shows a stronger applicability and optimization capability for different optimization scenarios. Compared with the GA-HH, which lacks a self-learning mechanism, the GA-SLHH shows enhanced optimization capabilities and adaptability to different weight cases. 

**Figure 13:**
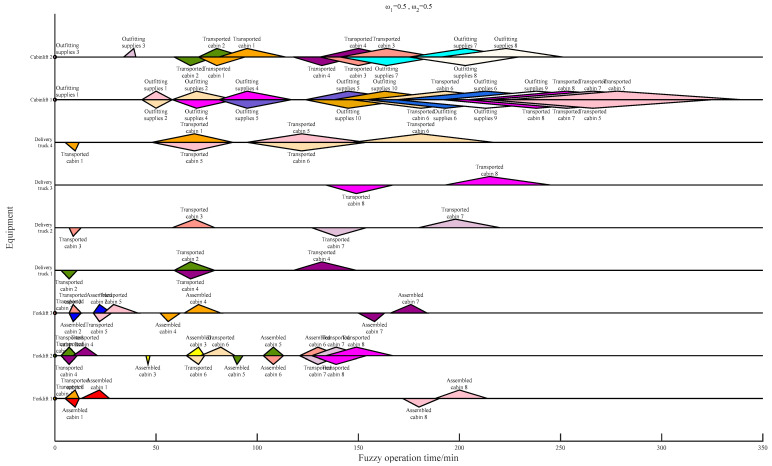
Scheduling scheme under $\omega_{1}=0.5,\omega_{2}=0.5$.

**Figure 14:**
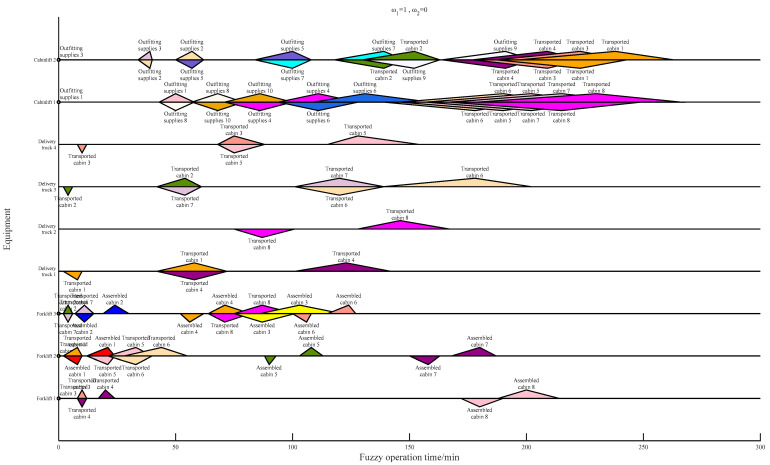
Scheduling scheme under $\omega_{1}=1,\omega_{2}=0$.

**Figure 15:**
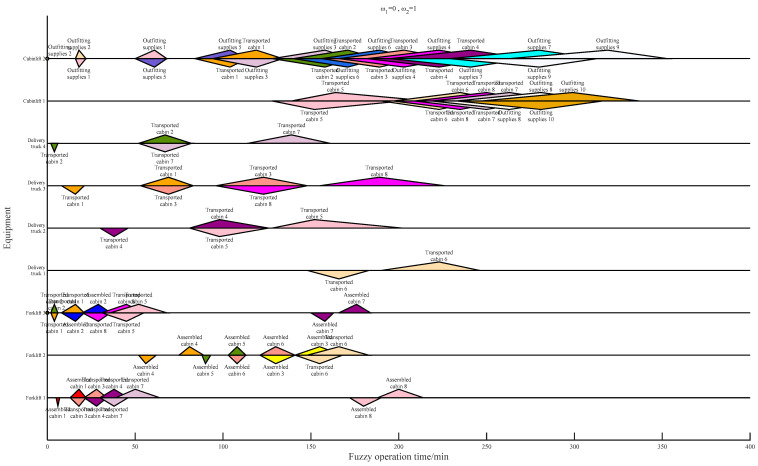
Scheduling scheme under $\omega_{1}=0,\omega_{2}=1$.

Table 13 shows the computational time for different meta-heuristics and hyper-heuristics. As a result of the hyper-heuristic framework, which can avoid a lot of judgment and repair operations of infeasible solutions, the computation time of the GA-SLHH is always less than that of the GA and PSO. The introduction of the self-learning mechanism incurs some additional computational costs; the computational time of the GA-HH remains slightly less than that of the GA-SLHH, which is also expected. As an application example, the Gantt charts of the results of the scheduling scheme with different weight settings are shown in Figure 13, Figure 14 and Figure 15.

## 6. Managerial Implications

This paper aims to solve the problem of collaborative scheduling for PMCU logistics in yard operations and transportation during the construction of domestic large cruise ships. The early or delayed completion of production activities is prevalent in Chinese cruise ship manufacturers. The construction of cruise ships is subject to strict plan constraints and require more robust activity management to control deviations from the actual schedule to plan. The constant-value scheduling scheme solved by conventional scheduling methods does not provide a complete measure of the early or delayed production activities. The fuzzy scheduling method proposed in this paper (i.e., the fuzzy mathematical model and GA-SLHH) converts the scheduling scheme from a constant value to a fuzzy time interval, which is able to evaluate and manage the production of the enterprise more efficiently and meets the needs of cruise ship construction projects. In addition, adding fuzzy processing time and fuzzy due date to the optimization model can generate more flexible and adaptive scheduling schemes, which can cope with the changes in and beyond the actual production in cruise ship manufacturers, thus enhancing the scheduling efficiency and robustness. 

Under further research of Industry 4.0 and intelligent ship manufacturing, Chinese cruise ship manufacturing enterprises should thoroughly consider the impact of each part of the cruise ship production process on production as much as possible when carrying out Just-In-Time (JIT) production. Especially for PMCU, which is a brand-new intermediate product for the shipbuilding industry, it is more important to arrange for the relevant departments to make rational production and transportation plans and guide the production operation effectively, to improve the efficiency of the intermediate process, reduce the cost of the enterprise, and enhance the competitiveness of the enterprise. According to the cruise ship manufacturer data estimation, the manufacturer currently takes 7–8 h to complete the PMCU logistic process. The on-site production department will set the delivery date within the required timeframe. The on-site production department will set the due date within the required time. However, the current scheduling approach is on-site and on-demand, and situations such as the temporary occupation of resources can cause delivery delays. The proposed algorithm in this study provides PMCU logistics scheduling from the overall project perspective. At the same time, enterprise managers set the weights to determine the optimization direction according to the actual situation of the project. For example, when the construction of a cruise ship’s cabin area is expedited, the optimization objective can be weighted with $\omega_{1}=1$. From the results of this paper, it can be seen that the maximum time required to complete all cabin logistics is reduced to 4.7 h, representing a 37% decrease in transportation time, thereby ensuring that on-site supply requirements for PMCUs are met. The rational scheduling approach can simultaneously improve the transportation efficiency of PMCUs and the degree of plan matching, which is very important for the future development of Chinese cruise ship manufacturing enterprises.

## 7. Conclusions

This paper mainly focuses on the collaborative scheduling problem of prefabricated cabin logistics in the production process of cruise ships and constructs a multi-objective fuzzy collaborative scheduling model for PMCU logistics. The objectives of this model are minimizing the maximum fuzzy makespan and the maximum fuzzy due date agreement index. We introduce a new genetic hyper-heuristic algorithm with a self-learning mechanism (GA-SLHH) to solve the problem. The performance of the GA-SLHH for the proposed problem are verified based on computational experiments, and the impact of the results for real-world production are analyzed. Our main conclusions are summarized below:

1. This research provides an in-depth analysis of the logistical operations for PMCUs. By characterizing the inherent uncertainties, the bidirectional operations, and the intersection of multiple tasks and equipment types, we have developed a multi-objective mathematical model that reflects the process of PMCU logistics. The two optimization objectives represent the efficiency and plan execution accuracy of PMCU logistics in cruise shipyard, respectively, which have higher practical application significance.
2. We propose a novel GA-SLHH to solve the model in this paper. The low-level heuristic is composed of 14 basic dispatching rules. The high-level strategy adopts genetic algorithms and self-learning mechanisms interacting together to drive the low-level heuristics. The self-learning mechanism introduced into the high-level strategy is more suitable for human decision-making characteristics and is more applicable to actual problems in shipyards.
3. The performance of the proposed GA-SLHH is validated through performance experiments and an enterprise case. The GA-SLHH shows a superior optimization performance compared to its competitors in the majority of performance experiment instances. In the enterprise case experiments, the GA-SLHH under different optimization weight settings all achieve the best objective function values, showing a better applicability to real-world problems.
4. Different from conventional scheduling methods, the GA-SLHH provides cruise shipyards with rational fuzzy scheduling schemes under different circumstances of the project. It is more beneficial for managers to adjust and control the production activities within PMCU logistics under different demands. In a certain application scenario, the scheduling scheme obtained using the methodology of this study can achieve an approximately 37% reduction in time.

The GA-SLHH proposed in this paper enhances the applicability of the models and methods in this paper to real-world problems, but it is lacking in considering the dynamic events that may occur in the logistics chain that cause large changes in the scheduling scheme. In future studies, dynamic factors in PMCU logistics, such as worker absence, equipment maintenance, and emergency arrival, will be investigated to further improve the applicability of the study. The suitable low-level heuristic operators and high-level strategies will be designed, so as to minimize the impact of the occurrence of dynamic events on the original scheduling scheme. Moreover, future work will focus on optimizing the framework of the GA-SLHH to enhance computational efficiency. This refinement is expected to expedite the processing of statistical probabilities, further elevating the model’s practical utility.

## Figures and Tables

**Table 1:** Summary of literature.

Papers	Objectives	Type	Method
Sugianto and Kim [54]	Delivery completion time	Single-objective	Iterated variable neighborhood search with rule-based heuristics
Song et al. [55]	Completion time	Single-objective	Hyper-heuristic based memetic algorithm
Soleimanian Gharehchopogh et al. [56]	Distance	Single-objective	Improved farmland fertility algorithm with hyper-heuristic
Li et al. [57]	Total completion time	Single-objective	Reinforcement learning-based hyper-heuristic
Duan et al. [58]	Tardiness, idle energy consumption, and makespan	Multi-objective	Genetic programming hyper-heuristic
Mahmud et al. [59]	Cost and sustainability rewards	Multi-objective	Self-adaptive hyper-heuristic
Cui et al. [60]	Makespan and resources	Multi-objective	Choice-function-based hyper-heuristic

**Table 2:** The operation rules of TFNs.

Operations on Fuzzy Time	Rules
+(sum)	$\tilde{C_{1}}+\tilde{C_{2}}=(c_{1}^{1}+c_{2}^{1},c_{1}^{2}+c_{2}^{2},c_{1}^{3}+c_{2}^{3})$
⋁(max)	$\tilde{C_{1}\;}⋁\tilde{C_{2}}=(c_{1}^{1}⋁c_{2}^{1},c_{1}^{2}⋁c_{2}^{2},c_{1}^{3}⋁c_{2}^{3})$
Ranking	Criterion 1. $C_{1}\left(\tilde{C}\right)=c^{1}+2c^{2}+c^{3}/4$
	Criterion 2. $C_{2}\left(\tilde{C}\right)=c^{2}$
	Criterion 3. $C_{3}\left(\tilde{C}\right)=c^{3}-c^{1}$

**Table 3:** Assumptions of PMCU logistics collaborative scheduling problem.

Assumptions	
1.	All transportation equipment is available at time 0.
2.	The release time of existing transport tasks is not 0.
3.	There are a variety of types of transport tasks to be transported, and the transport stages of each type of task are different.
4.	There is no waiting time for the same transport equipment for adjacent tasks.
5.	The buffer capacity between each stage is unlimited; that is, there is no blocking constraint.
6.	Once the transport task is carried out on transport equipment, no interruption is allowed until the completion of the stage of transport.
7.	The preparation time and return time of the equipment have been considered in task transportation time.
8.	The same equipment can only process one task at a time.

**Table 4:** Definitions and descriptions of symbols.

Symbols	Meaning
Indices	Meaning
i,j	Task index i,j, i,j=1,2,…,n; n is the total number of transportation tasks in PMCU logistics
s	Transport stage index s$,\;s=1,2,\ldots ,n_{s}$; $n_{s}$ is the total number of stages in PMCU logistics
k	Equipment index k$,\;k=1,2,\ldots ,n_{m}$; $n_{m}\;$is the total amount of equipment in PMCU logistics
Parameters	Meaning
$\tilde{D_{i}}$	The fuzzy due date of task i
$\tilde{P_{iks}}$	The fuzzy time of task i required for transportation by equipment k in stage s
$\tilde{R_{i}}$	The fuzzy release time of task i
$Y_{is}$	Binary parameter. If task i $\;\mathrm{goes}\;\mathrm{through}\;\mathrm{the}\;\mathrm{stage}\;s,\;Y_{is}=1$ $;\;\mathrm{otherwise},\;Y_{is}=0$
M	Large positive number
Variables	Meaning
$\tilde{C_{is}}$	The fuzzy completion time of stage s of task i
$\tilde{B_{is}}$	The fuzzy beginning time of stage s of task i
$\tilde{C_{i}}$	The fuzzy completion time of the last stage of task i
$\tilde{C_{max}}$	The maximum fuzzy makespan of PMCU logistics
$\bar{AI}$	The average fuzzy due date agreement index of PMCU logistics
$X_{iks}$	If task i is transported by equipment k in stage s $,\;X_{iks}=1$ $;\;\mathrm{otherwise},\;X_{iks}=0$
$Z_{ijks}$	If tasks i and j are transported by equipment k in stage s, and i$\;\mathrm{is}\;\mathrm{transported}\;\mathrm{before}\;j,Z_{ijks}=1$$;\;\mathrm{otherwise},\;Z_{ijks}$= 0

**Table 5:** Dispatching rules.

No.	Dispatching Rule	Description
1	FIFO	First in, first out
2	SPT	Shortest processing time
3	LPT	Longest processing time
4	LWKR	Least work remaining
5	MWKR	Most work remaining
6	SPTswm [64]	Shortest average processing time
7	LPTswm [64]	Longest average processing time
8	SDT	Shortest processing time as a percentage of total time
9	LDT	Longest processing time as a percentage of total time
10	SDR	Shortest processing time as a percentage of the remaining time
11	LDR	Longest processing time as a percentage of the remaining time
12	FRO	Fewest remaining operations
13	LRO	Most remaining operations
14	Random	Random selection

**Table 6:** LLH-solving process of the scheme.

Step	Description
Step 1	Parameters initialization, transport stage s=1, dispatching rule number n=1.
Step 2	$\mathrm{Determine}\;\mathrm{the}\;\mathrm{set}\;T_{s},\;$which is the set of tasks that need to be processed in stage s and the set of equipment.
Step 3	Select the earliest idle equipment from the set of idle equipment.
Step 4	Select the n−th dispatching rule in LLH and determine the current task i to be processed according to the dispatching rule.
Step 5	Updating the completion time of task i and the earliest idle time of equipment k,n=n+1.
Step 6	$\mathrm{If}\;\mathrm{set}\;T_{s}\;$is empty, and the current stage s is not the last stage, s=s+1 and execute Step 2. Otherwise, execute Step 7.
Step 7	Generate a scheduling scheme. Calculate the fitness corresponding to the current scheduling scheme based on the fitness calculation function and pass it to the HLS.

**Table 7:** Parameters of algorithms.

Algorithms	Type	Parameters
GA	Metaheuristic	Crossover rate cr=0.8 , mutation rate mr=0.1
PSO		Inertia weight ω=0.85 $,\;\mathrm{learning}\;\mathrm{factor}\;c_{1}=c_{2}=2$
GA-SLHH	Hyper-heuristic	Crossover rate cr=0.8 , mutation rate mr=0.1
GA-HH		Crossover rate cr=0.8 , mutation rate mr=0.1
FIFO	Dispatching rule	None
SPT		
MWKR		
FRO		

**Table 8:** Average values of 30 times for benchmark instances.

Benchmark Instances		Metaheuristic		Hyper-Heuristic		Dispatching Rule			
		GA	PSO	GA-SLHH	GA-HH	FIFO	SPT	MWKR	FRO
Instance 1	$F_{avg}$	1.301	1.111	0.545	0.546	0.812	0.696	2.262	0.681
	$f_{1}$	48.09	49.13	36.99	37.05	40.75	35.5	53.75	41
	$f_{2}$	0.646	0.596	0.944	0.943	0.824	0.817	0.230	0.761
Instance 2	$F_{avg}$	1.175	1.261	0.640	1.069	1.039	1.001	1.967	1.206
	$f_{1}$	66.90	65.51	51.28	55.39	54.75	65.75	63	63.5
	$f_{2}$	0.401	0.354	0.547	0.634	0.655	0.745	0.305	0.565
Instance 3	$F_{avg}$	1.615	1.729	0.845	0.969	0.928	0.941	2.197	1.282
	$f_{1}$	74.05	75.15	55.56	60.63	62	67.25	68.75	68.25
	$f_{2}$	0.413	0.383	0.642	0.768	0.838	0.828	0.272	0.638
Instance 4	$F_{avg}$	1.440	1.456	0.805	0.935	0.817	0.812	2.221	0.914
	$f_{1}$	60.30	61.35	46.97	50.56	47.5	50	60.5	49.75
	$f_{2}$	0.447	0.454	0.643	0.754	0.863	0.898	0.263	0.752

**Table 9:** Parameters for generating cases.

Case	Number of Tasks	Number of Equipment	Number of Operations per Task	Processing Time of per Operation
Case 1	4	2	[1, 3]	[1, 5]
Case 2	4	4	[1, 3]	[1, 5]
Case 3	6	4	[3, 5]	[2, 7]
Case 4	6	6	[3, 5]	[2, 7]
Case 5	8	6	[4, 6]	[5, 9]
Case 6	10	6	[4, 6]	[6, 13]
Case 7	10	10	[5, 7]	[7, 20]
Case 8	10	10	[5, 10]	[5, 25]
Case 9	15	5	[4, 6]	[1, 10]
Case 10	15	8	[5, 7]	[10, 15]
Case 11	15	8	[5, 10]	[1, 10]
Case 12	20	5	[5, 10]	[5, 13]
Case 13	20	8	[5, 7]	[5, 13]
Case 14	20	10	[5, 7]	[10, 30]
Case 15	30	10	[8, 10]	[10, 30]

**Table 10:** Statistical values of algorithms.

Case	Metaheuristic				Hyper-Heuristic				Dispatching Rule							
	GA		PSO		GA-SLHH		GA-HH		FIFO		SPT		MWKR		FRO	
	mean	std.	mean	std.	mean	std.	mean	std.	mean	std.	mean	std.	mean	std.	mean	std.
Case 1	0.788	0.065	0.789	0.069	**0.702**	0.012	0.874	0.081	1.759	0.000	1.905	0.000	4.533	0.000	1.004	0.000
Case 2	0.512	0.015	0.517	0.021	**0.504**	0.000	0.534	0.025	0.641	0.000	0.524	0.000	0.924	0.000	0.641	0.000
Case 3	0.932	0.066	0.924	0.113	**0.908**	0.042	1.089	0.084	1.192	0.000	1.081	0.000	3.054	0.000	1.287	0.000
Case 4	0.657	0.032	0.652	0.026	**0.609**	0.023	0.661	0.021	0.693	0.000	0.644	0.000	0.860	0.000	0.819	0.000
Case 5	1.138	0.096	1.150	0.103	1.088	0.035	1.223	0.050	1.323	0.000	**1.062**	0.000	1.546	0.000	1.306	0.000
Case 6	1.567	0.146	1.608	0.205	1.560	0.049	1.774	0.098	2.071	0.000	**1.537**	0.000	3.522	0.000	1.982	0.000
Case 7	1.067	0.124	1.053	0.085	1.037	0.019	1.114	0.035	1.179	0.000	**1.001**	0.000	1.580	0.000	1.251	0.000
Case 8	1.293	0.107	1.202	0.160	**0.888**	0.039	0.951	0.023	1.234	0.000	1.120	0.000	1.624	0.000	1.625	0.000
Case 9	6.784	2.343	6.717	2.174	**6.277**	0.582	9.829	1.741	60.737	0.000	17.732	0.000	694.691	0.000	20.927	0.000
Case 10	3.301	0.457	3.216	0.569	**2.747**	0.077	3.038	0.125	3.093	0.000	2.798	0.000	6.236	0.000	3.298	0.000
Case 11	2.998	0.432	3.125	0.582	**1.817**	0.080	2.143	0.154	2.203	0.000	2.940	0.000	4.601	0.000	2.219	0.000
Case 12	20.983	4.017	20.864	4.154	**14.122**	0.662	16.734	1.077	25.720	0.000	26.400	0.000	57.592	0.000	26.205	0.000
Case 13	8.556	1.851	9.403	2.387	**5.138**	0.220	5.994	0.479	11.074	0.000	11.070	0.000	24.868	0.000	9.182	0.000
Case 14	4.896	1.054	4.744	1.031	**3.756**	0.150	4.393	0.275	4.901	0.000	6.074	0.000	30.239	0.000	5.515	0.000
Case 15	56.615	25.364	59.560	31.099	**22.422**	2.206	37.503	6.097	55.684	0.000	95.365	0.000	118.121	0.000	43.898	0.000

**Table 11:** Fuzzy transport time and due date of PMCU logistics equipment.

Task	No.	Assembly Completion Time	Fuzzy due Date	Fuzzy Transportation Time								
				Forklift			Delivery Truck				Cabin Lift	
				1	2	3	1	2	3	4	1	2
Assembled PMCU	1	(5,6,7)	(13,15)	(8,12,15)	(10,13,14)	(12,14,15)	-	-	-	-	-	-
	2	(8,9,10)	(17,20)	(10,16,20)	(11,13,15)	(12,13,15)	-	-	-	-	-	-
	3	(45,46,47)	(53,59)	(15,18,20)	(20,25,27)	(11,16,20)	-	-	-	-	-	-
	4	(52,56,62)	(60,63)	(16,23,25)	(23,25,27)	(12,15,20)	-	-	-	-	-	-
	5	(88,90,93)	(110,115)	(14,15,19)	(15,18,20)	(13,16,19)	-	-	-	-	-	-
	6	(100,106,108)	(120,130)	(17,20,25)	(18,22,28)	(15,18,19)	-	-	-	-	-	-
	7	(150,158,163)	(176,182)	(15,20,25)	(18,22,24)	(16,18,21)	-	-	-	-	-	-
	8	(172,180,190)	(191,203)	(16,20,24)	(13,17,20)	(17,22,25)	-	-	-	-	-	-
PMCU awaiting transportation	1	-	(63,70)	(5,10,12)	(2,8,10)	(6,12,15)	(40,50,62)	(53,65,73)	(45,53,62)	(43,59,76)	-	(10,15,20)
	2	-	(75,80)	(4,8,15)	(3,7,11)	(2,4,6)	(56,60,68)	(50,58,66)	(40,50,55)	(50,63,76)	-	(12,13,15)
	3	-	(160,172)	(8,10,12)	(5,7,10)	(7,9,13)	(58,66,73)	(51,60,66)	(43,54,65)	(60,65,76)	-	(10,14,19)
	4	-	(92,103)	(9,10,12)	(6,8,10)	(10,11,13)	(59,65,70)	(51,60,80)	(56,63,65)	(42,55,63)	-	(13,18,21)
	5	-	(109,115)	(11,13,15)	(9,12,16)	(5,7,13)	(43,56,69)	(47,54,76)	(35,56,60)	(47,53,66)	(11,12,15)	-
	6	-	(225,246)	(8,10,13)	(7,11,15)	(8,10,13)	(42,57,63)	(45,54,66)	(38,58,63)	(52,59,63)	(7,12,15)	-
	7	-	(235,250)	(9,12,18)	(6,9,13)	(5,7,9)	(42,56,63)	(53,59,66)	(59,66,78)	(62,72,79)	(10,13,17)	-
	8	-	(247,260)	(4,5,10)	(7,10,13)	(11,16,19)	(46,56,63)	(53,59,66)	(59,66,78)	(62,72,79)	(12,15,17)	
Outfitting supplies	1	-	(65,75)	-	-	-	-	-	-	-	(43,50,58)	(34,43,46)
	2	-	(70,83)	-	-	-	-	-	-	-	(15,20,31)	(16,18,22)
	3	-	(110,120)	-	-	-	-	-	-	-	(23,25,28)	(34,39,40)
	4	-	(110,120)	-	-	-	-	-	-	-	(23,25,28)	(16,20,22)
	5	-	(156,170)	-	-	-	-	-	-	-	(43,50,58)	(34,43,46)
	6	-	(164,180)	-	-	-	-	-	-	-	(15,20,31)	(16,18,22)
	7	-	(180,190)	-	-	-	-	-	-	-	(23,25,28)	(34,39,40)
	8	-	(176,193)	-	-	-	-	-	-	-	(14,18,21)	(16,20,22)
	9	-	(208,212)	-	-	-	-	-	-	-	(23,25,28)	(34,39,40)
	10	-	(245,260)	-	-	-	-	-	-	-	(14,18,21)	(16,20,22)

**Table 12:** Case verification results with different weights.

Algorithms	Type	Weight	$F_{avg}$	$F_{best}$	$F_{worst}$
GA	Metaheuristic	$\omega_{1}=0.5,\omega_{2}=0.5$	7.386	4.836	10.023
		$\omega_{1}=1,\omega_{2}=0$	0.466	0.336	0.634
		$\omega_{1}=0,\omega_{2}=1$	8.567	6.664	10.796
PSO		$\omega_{1}=0.5,\omega_{2}=0.5$	7.706	5.279	9.514
		$\omega_{1}=1,\omega_{2}=0$	0.634	0.281	0.841
		$\omega_{1}=0,\omega_{2}=1$	7.962	5.257	11.568
GA-SLHH	Hyper-heuristic	$\omega_{1}=0.5,\omega_{2}=0.5$	2.813	2.336	3.510
		$\omega_{1}=1,\omega_{2}=0$	0.253	0.210	0.287
		$\omega_{1}=0,\omega_{2}=1$	5.528	4.479	6.645
GA-HH		$\omega_{1}=0.5,\omega_{2}=0.5$	3.927	2.692	5.181
		$\omega_{1}=1,\omega_{2}=0$	0.319	0.271	0.409
		$\omega_{1}=0,\omega_{2}=1$	7.102	5.137	9.444
FIFO	Dispatching rule	$\omega_{1}=0.5,\omega_{2}=0.5$	-	4.154	-
		$\omega_{1}=1,\omega_{2}=0$	-	0.216	-
		$\omega_{1}=0,\omega_{2}=1$	-	8.093	-
SPT		$\omega_{1}=0.5,\omega_{2}=0.5$	-	3.531	-
		$\omega_{1}=1,\omega_{2}=0$	-	0.313	-
		$\omega_{1}=0,\omega_{2}=1$	-	6.749	-
MWKR		$\omega_{1}=0.5,\omega_{2}=0.5$	-	6.587	-
		$\omega_{1}=1,\omega_{2}=0$	-	0.217	-
		$\omega_{1}=0,\omega_{2}=1$	-	12.958	-
FRO		$\omega_{1}=0.5,\omega_{2}=0.5$	-	4.226	-
		$\omega_{1}=1,\omega_{2}=0$	-	0.418	-
		$\omega_{1}=0,\omega_{2}=1$	-	8.033	-

**Table 13:** Computational times of different algorithms.

Algorithm	Computational Time (Unit: Seconds)		
	$\omega_{1}=0.5,\omega_{2}=0.5$	$\omega_{1}=1,\omega_{2}=0$	$\omega_{1}=0,\omega_{2}=1$
GA	25.869	24.617	25.249
PSO	26.429	27.052	26.888
GA-SLHH	16.754	16.614	17.217
GA-HH	11.659	12.112	12.597

## Data Availability

The data are contained within the article.
